# Supplementary material for: KSK-74: Dual Histamine H3 and Sigma-2 Receptor Ligand with Anti-Obesity Potential
Source: Int J Mol Sci. 2022 Jun 24;23(13):7011. doi: 10.3390/ijms23137011 (PMC9266460; doi:10.3390/ijms23137011)
Supplement: Supplementary file 1 [file ijms-23-07011-s001.zip › Supplement File S1 - Structure and purity confirmation data of compounds KSK.pdf]

Structure and purity confirmation data of compounds KSK-60 and KSK-74

KSK60:

^{1}H NMR (DMSO-d_6, 300 MHz) δ ppm: 8.2-8.3 (m, 2H, *J*=7.6 Hz, 4Pyr-2,6H), 7.9-7.9 (m, 2H, *J*=8.8 Hz, Ph-3,5H), 7.1-7.2 (m, 2H, *J*=7.0 Hz, Ph-2,6H), 7.0-7.0 (m, 2H, *J*=8.8 Hz, 4Pyr-3,5H), 4.04 (br t, 2H, *J*=6.2 Hz, CH_2-O), 3.68 (br s, 4H, Pip-3,5H), 2.95 (q, 2H, *J*=7.2 Hz, CO-CH_2-CH_3), 2.71 (br s, 4H, Pip-2,6H), 2.49 (m, 3H, CO-CH_2-CH_3), 1.7-1.8 (m, 2H, NCH_2), 1.3-1.6 (m, 6H, $CH_2CH_2CH_2CH_2$), 1.04 (m, 2H, *J*=7.0 Hz, $CH_2CH_2CH_2CH_2$). ^{13}C NMR (DMSO-d_6): 199.24, 162.83, 130.54, 129.85, 114.68, 108.19, 68.18, 56.99, 51.71, 45.03, 33.02, 31.22, 28.85, 26.70, 25.66, 8.77. LC–MS: purity 100.00% t_R = 3.43, (ESI) *m/z* $[M+H]^+$ 396.22.

KSK74:

^{1}H NMR (DMSO-d_6, 500 MHz) δ ppm: 8.24 (br d, 2H, *J*=6.9 Hz, 4Pyr-2,6H), 7.88 (br d, 2H, *J*=8.6 Hz, Ph-3,5H), 7.15 (br d, 2H, *J*=6.9 Hz, Ph-2,6H), 6.97 (br d, 2H, *J*=8.6 Hz, 4Pyr-3,5H), 4.01 (br t, 2H, *J*=6.3 Hz, CH2-O), 3.71 (br s, 4H, Pip-3,5H), 2.93 (q, 2H, *J*=7.4 Hz, CO-CH_2-CH_3), 2.80 (br s, 4H, Pip-2,6H), 2.60 (br t, 2H, *J*=7.4 Hz, NCH_2), 1.7-1.7 (m, 2H, $CH_2CH_2CH_2CH_2CH_2CH_2$), 1.51 (br s, 2H, $CH_2CH_2CH_2CH_2CH_2CH_2$), 1.38 (br s, 2H, $CH_2CH_2CH_2CH_2CH_2CH_2$), 1.27 (br s, 6H, $CH_2CH_2CH_2CH_2CH_2CH_2$), 1.02 (br t, 3H, J=7.2 Hz, CO-CH_2-CH_3). ^{13}C NMR (DMSO-d_6): 199.34, 162.93, 162.72, 157.10, 141.08, 130.63, 129.93, 114.77, 108.51, 68.32, 56.28, 50.82, 50.81, 31.30, 29.01, 26.67, 25.89, 24.10, 14.42, 8.48. LC–MS: purity 100.00% t_R = 4.03, (ESI) *m/z* $[M+H]^+$ 424.34.
