# Supplementary figures and images for: KSK-74: Dual Histamine H3 and Sigma-2 Receptor Ligand with Anti-Obesity Potential
Source: Int J Mol Sci. 2022 Jun 24;23(13):7011. doi: 10.3390/ijms23137011 (PMC9266460; doi:10.3390/ijms23137011)

Standard curves.

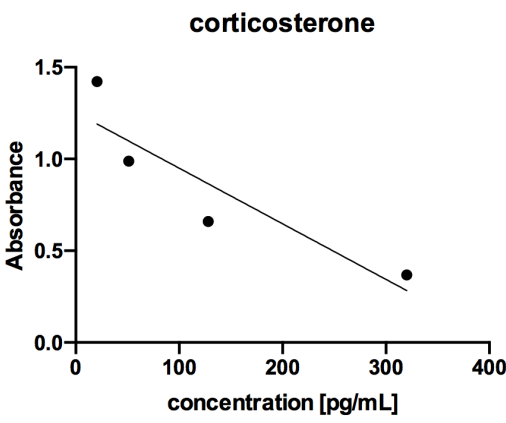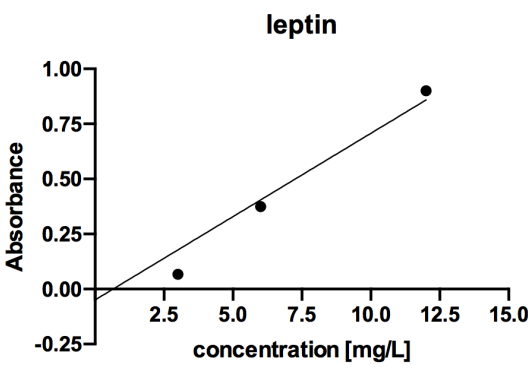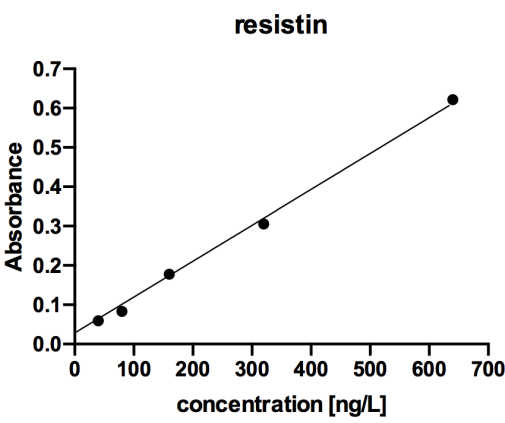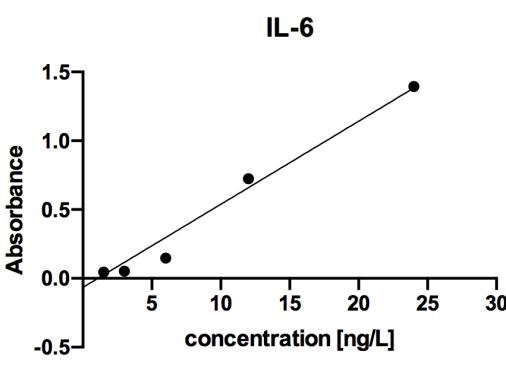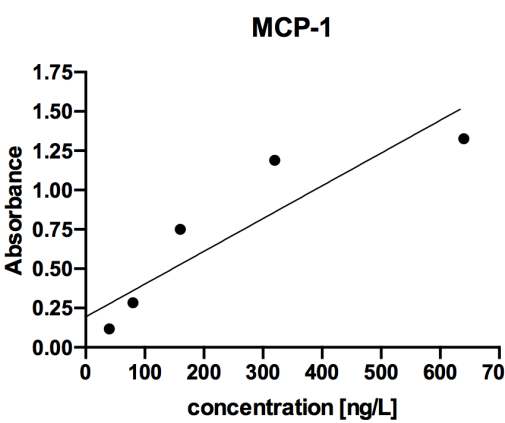

Supplement: Supplementary file 1 [file ijms-23-07011-s001.zip › Supplement File S2 - Standard curves.pdf]
